# Primary thyroid squamous cell carcinoma with severe respiratory stenosis and endotracheal invasion: a case report with literature review

**DOI:** 10.3389/fmed.2025.1631714

**Published:** 2025-07-25

**Authors:** Xueliang Liu, Shang Ma, Yuxin Zheng, Zeming Xu, Hui Hao, Hongyan Ma, Qian Xu

**Affiliations:** ^1^Department of Thyroid and Breast Surgery, Cangzhou People’s Hospital, Cangzhou, China; ^2^Department of Oncology, Cangzhou People’s Hospital, Cangzhou, China; ^3^Department of Pathology, Cangzhou People’s Hospital, Cangzhou, China; ^4^Department of Anesthesiology, Cangzhou People’s Hospital, Cangzhou, China

**Keywords:** thyroid cancer, primary squamous cell carcinoma, thyroid, case report, respiratory stenosis

## Abstract

**Introduction:**

Primary squamous cell carcinoma of the thyroid (PSCCT) is a rare and highly aggressive malignant tumor with a poor prognosis. Although surgery, chemotherapy and other treatment methods have been reported, the current treatment modality has not reached a consensus. This study discusses the diagnosis and treatment of a case of PSCCT with severe respiratory stenosis and endotracheal invasion and reviews the relevant literature. We report the disease of rapidly enlarging mass leading to asphyxiation to raise clinicians’ awareness of this condition.

**Case presentation:**

We report a 76-year-old woman presenting with an enlarging right thyroid mass accompanied by severe dyspnea and hoarseness. Computed tomography (CT) scan disclosed a large solid heterogenous nodule with calcification in the right thyroid lobe and prominent adjacent lymph nodes. PSCCT was confirmed by postoperative histopathology and immunohistochemistry. Thyroidectomy with partial tracheectomy and tracheostomy was performed to relieve the patient’s dyspnea. The patient has been discharged after receiving post-operative supportive care.

**Conclusion:**

Clinicians should pay attention to the rapidly enlarging neck mass as it may cause asphyxiation and avoid the loss of treatment opportunities.

## Introduction

Primary squamous cell carcinoma of the thyroid (SCCT) is a very rare entity that accounts for <1% of all primary thyroid cancers and has extremely poor prognosis (1). 117 SCCT cases were reported in the medical literature, and nearly half of the cases (56) have been reported in Asia, with the majority reported in Japan (2). PSCCT is characterized by a highly aggressive tumor with a poor prognosis and survival usually less than 1 year (3). It usually presents as a rapidly expanding anterior neck mass with involvement of the surrounding tissues and often leads to dyspnea and hoarseness (4). The low incidence of PSCCT contributes to the complexity of understanding its pathogenesis, diagnosis, clinical presentation, and treatment strategies. Due to rapid mass growth, the diagnosis of PSCCT is usually challenging, it can easily be misdiagnosed as acute thyroiditis or other thyroid tumor (3). Furthermore, the crucial step is ruling out secondary SCC metastasis from other primary sites, as treatment and prognosis of SCC varies greatly (5). As surgery is the primary treatment, early diagnosis is critical to ensure that radical resection remains possible in cases of locally advanced disease (2, 6). Furthermore, an SCC mass can compress or even infiltrate the trachea, causing airway obstruction; tracheostomy may then be performed to relieve dyspnea if necessary (3, 5, 7). Radiotherapy, chemotherapy, and targeted therapies can also be used as adjuvant treatments, although in some cases these treatments are not sensitive to patients.

## Case presentation

**Figure 1:**
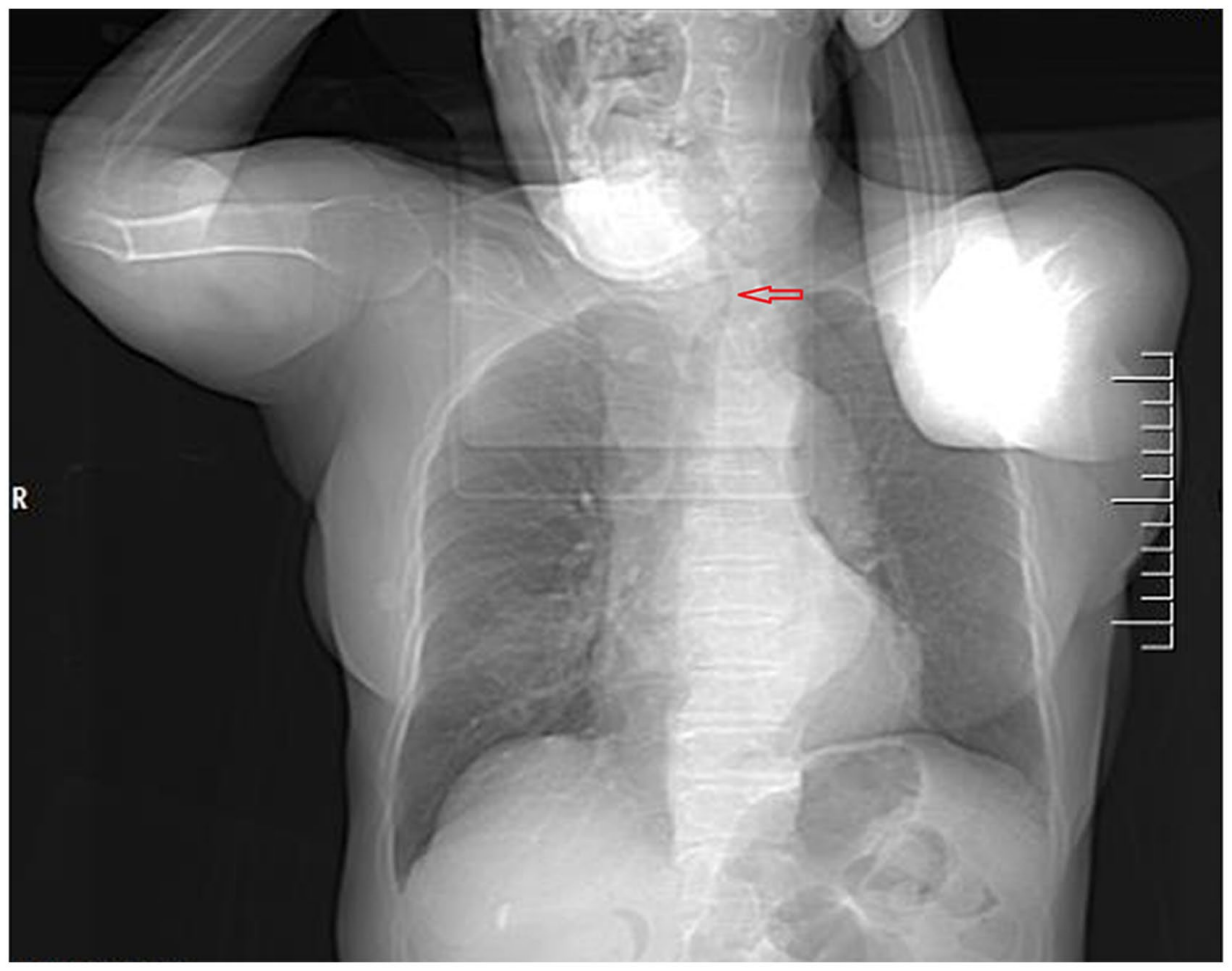
Computed tomography of the chest and neck revealed tracheal stenosis (arrow) and left-sided deviation (coronal planes).

**Figure 2:**
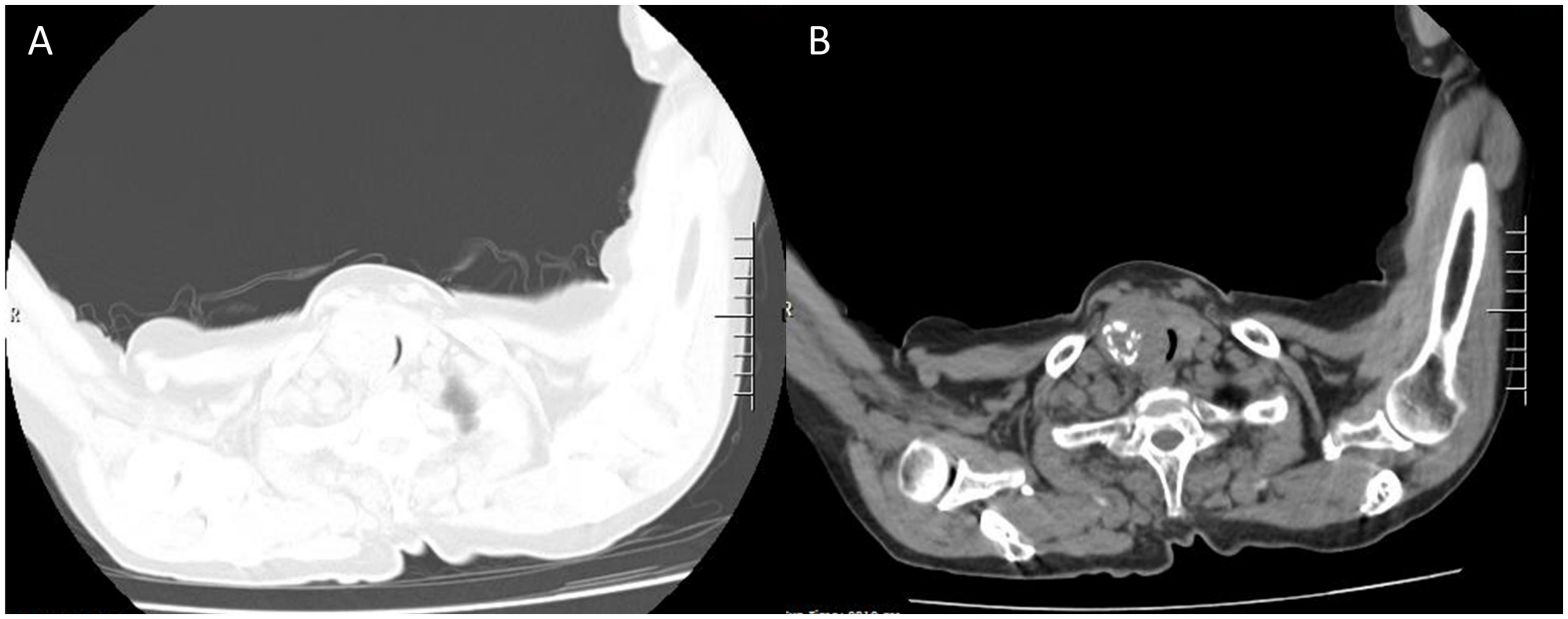
The same level of lung and mediastinal window CT scan show heterogeneously solid mass sized about 5.5 × 4 cm **(A)** in the right thyroid gland with calcification **(B)** and indistinct boundaries, tracheal stenosis (2 mm) and left deviation (**A**, lung window; **B**, mediastinal window).

**Figure 3:**
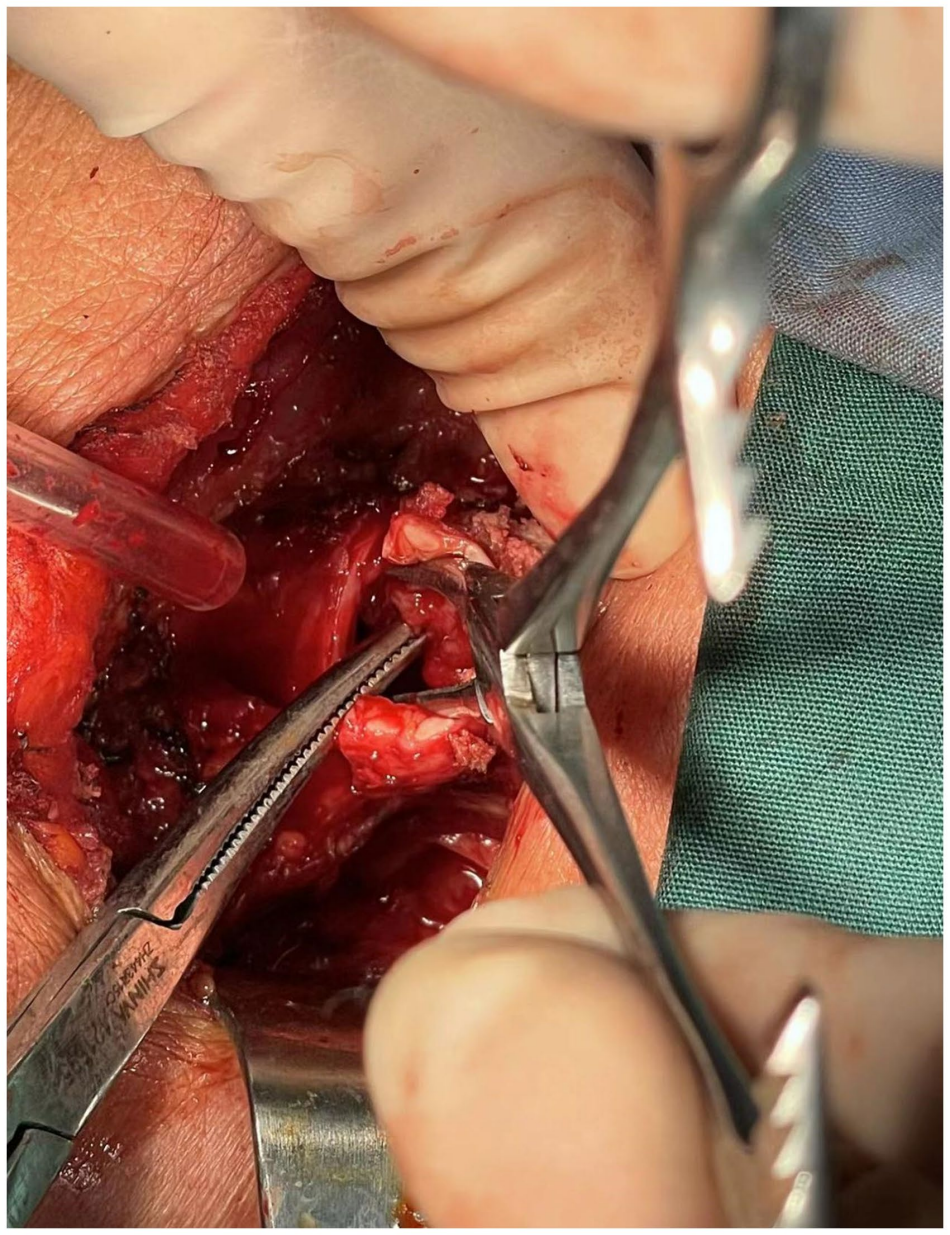
The thyroid carcinoma has invaded into the trachea.

A 76-year-old female presented to the Respiratory and Critical Care Medicine Department of our hospital with a gradually enlarging neck mass for 2 months, accompanied by progressive worsening of cough, sputum production, and dyspnea. Medical history: The patient had a left upper lobe lung resection for adenocarcinoma of the lung 3 years ago. The laboratory test results were normal: TSH:3.22 μIU/mL (reference range: 0.56–5.91 μIU/L); FT3: 5.16 pmol/L (reference range: 3.28–6.47 pmol/L); Free thyroxine: 14.49 pmol/L (reference range: 7.64–16.03 pmol/L). The tumor marker CA19-9 was 37.5 U/mL (reference range, 0–27 U/mL), and the results of other markers (CEA, AFP, CA125, CA15-3, CA7-24, SCC, ProGRP, Ferrit, and calcitonin) were all within the normal range. Neck CT enhanced scan: large lesion in the right lobe of the thyroid gland, with patchy calcification within, significant compression of the trachea and lateral displacement of the trachea to the left, narrowing of the trachea (thyroid tumor invasion of the trachea, the narroworst position of the airway is 2 mm) (Figures 1, 2). No obvious metastasis to the lymph nodes is seen, and no distant metastasis is noted. Flexible fiberoptic nasolaryngoscopy examination was not performed on the patient because of the poor condition and the potential risk of inducing asphyxia. For the past 3 days, the patient’s breathing difficulty has been gradually worsening, with occasional episodes of asphyxiation. The patient was transferred to the Thyroid and Neck Surgery Department for treatment. Physical examination revealed a palpable nodule measuring approximately 5 cm in diameter in the right thyroid region, with hard texture, poor mobility, and mild tenderness. No obvious enlarged lymph nodes were palpable in the neck. After consulting with Multi-Disciplinary Treatment, a total thyroidectomy with partial tracheectomy and tracheostomy was performed under general anesthesia. Due to the patient’s airway obstruction, it was not possible to perform a routine anesthesia. The patient was placed in a semi-sitting position and maintained spontaneous breathing with an endotracheal tube while being appropriately sedated and analgesic. The tip of the endotracheal tube was inserted 4 cm below the vocal cords, but it was unable to pass through the narrowed area. In order to facilitate endotracheal intubation, we performed regional airway anesthesia before the operation. The endotracheal tube was secured in place, anesthetic induction drugs were administered, and high-frequency ventilation was used to maintain breathing. Due to tumor invasion of the trachea (Figure 3), after the entire thyroid gland was removed, an incision was made in the trachea to insert a tracheal tube, and breathing was restored after the operation. The patient was then transferred to the ward.

**Figure 4:**
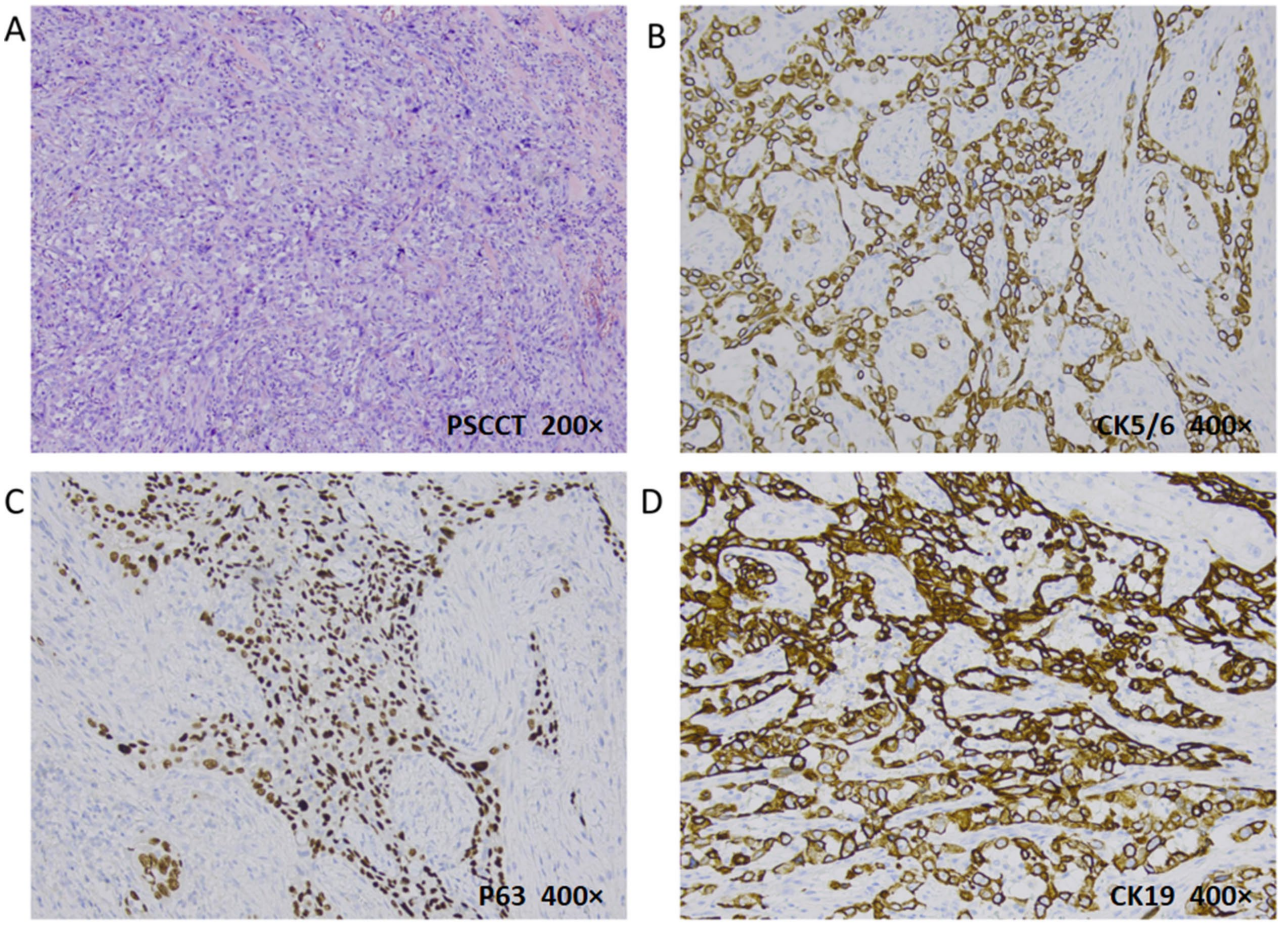
Histopathologic evaluation revealed PSCCT (H&E, 200×): poorly differentiated tumor cells and typical nuclear heterogeneous cells **(A)**. PSCC immunohistochemistry: CK5/6 **(B)** and P63 **(C)** were positive (deeply membranes and nuclei stained respectively), indicating the source of squamous cells; CK19 was positive for membranes stained **(D)** (400×).

Intraoperative frozen pathology: malignant thyroid tumor invades the tracheal muscle, right laryngeal nerve, esophageal muscle outside, and trachea. Postoperative pathology: Primary low-differentiated squamous cell carcinoma of the thyroid, size 5.5*4*2.5 cm, invading the fibrous and adipose tissue around the thyroid. Intrathoracic mass: Squamous cell carcinoma of the thyroid. Immunohistochemistry: Ki67 (40%+), TTF-1 (−), NapsinA (−), HNF1β (−), CD56 (−), CgA (−), SYN (−), CK19 (+), Calponin (−), T g (−), CK (+), Vimentin (+), Galectin-3 (+), P40 (positive), P63 (+), CK5/6 (+) (Figure 4). In order to facilitate recovery, appropriate fasting and fluid restriction measures, as well as anti-inflammatory and expectorant supportive therapies were given, and the patient’s condition stabilized. The patient was very satisfied with the relief of airway obstruction, but was not active in the postoperative adjuvant treatment. In fact, for locally advanced PSCCT, the first consideration for patients may be the quality of life, such as relieving airway obstruction and maintaining unobstructed breathing. The patient’s fear of asphyxia is greater than their concern about the impact of tumor on their own survival time.

## Timeline

The patient was hospitalized with enlarging neck mass and 3-year history of lung adenocarcinoma. The operation was performed on the 5th day after laboratory and imaging examinations and preoperative evaluations. Postoperative pathology confirmed the diagnosis as PSCCT, and the patient was discharged on the 17th day after the operation (Supplementary Figure S1).

## Discussion

PSCCT is a malignant tumor with extremely rare incidence which typically has a poor prognosis. Typically arising in older adults, the mean age of diagnosis for PSCCT patients was 68 (8). The case of 76-year-old female we reported is a PSCCT coexisting with lung adenocarcinoma and is easily misdiagnosed as metastatic carcinoma of lung, which has not been previously reported in literature. Therefore, it suggests that the possibility of PSCCT should still be considered when the thyroid mass is rapidly enlarged in the presence of other primary tumors.

The cause and the origin of PSCCT are highly controversial because thyroid gland itself does not have a squamous epithelial component in its anatomy. There are three common theories: dedifferentiation of other thyroid carcinoma, such as Papillary thyroid carcinoma (PTC) and Follicular thyroid carcinoma (FTC); the residual embryonic (it assumes that during development the embryonic remnants of squamous epithelial cells (e.g., thymic epithelium, thyroglossal duct) evolve into squamous epithelial cells) and squamous epithelial metaplasia theory (PSCCT could develop from the thyroid follicular epithelium associated with chronic inflammation) (2).

As squamous cell carcinoma of thyroid is highly aggressive, the poor prognosis of patients is mainly due to failure to diagnose and early treatment. It requires a comprehensive approach involving clinical symptoms, imaging, pathology, and immunohistochemistry to confirm the diagnosis. Secondary squamous cell carcinoma of the thyroid (SSCCT) is a type of malignant tumor that metastasizes from the larynx, esophagus, lung, and other primary sites. Since SSCCT is more common than PSCCT and the treatments vary widely, it is critical to distinguish between them for accurate treatment. Immunohistochemistry plays an important role in diagnosis of PSCCT, such as PAX-8 (paired box protein 8) marker positive (5, 9). TTF-1 and CK7 positive are helpful in diagnosing PSCCT as distinct from SSCCT (2).

A study (8) has shown that PSCCT had a larger tumor size (mean size 5.35 cm) than other types of thyroid carcinoma, and with more frequent positive margins and metastasis. CT (computed tomography) can show thyroid tumors and their relationship with the surrounding tissues. CT scan of the neck, chest, and abdomen can also be used to rule out SSCCT as the primary source.

Most PSCCT patients present with neck swelling as a rapidly enlarging anterior neck mass (69.6% tumor size was >4.0 cm) (10). As the tumor grows and invades the muscles and soft tissues, patient usually complains of neck discomfort or pain (11, 12). Dyspnea, dysphagia and hoarseness are common symptoms in cases as the advanced disease invades the trachea, esophagus and the recurrent laryngeal nerve (13–16). The associated symptoms are always followed by the carcinoma, such as weight loss (2). It must be emphasized that emergency tracheostomy usually needs to be performed because most patients with advanced disease succumb to airway obstruction (9, 17, 18). For this patient, dyspnea continued to worsen and there were frequent episodes of asphyxia, relief of airway obstruction is the first step that needs to be performed.

Early detection and diagnosis are important and possible for curative surgical resection (10). However, early diagnosis is difficult due to the lack of typical clinical features, late phase tumor always developed extensive local tissue infiltrated and metastasis with poor prognosis when detected. Although R0 resection was achieved in only 18.8% of all ThySCC patients (8), surgery remains to be considered an primary effective option to reduce tumor burden and relieve symptoms of local invasion (2, 19). As study shown, whether complete tumor resection or not (R0 or R1/R2), surgery was associated with favorable prognosis (8, 20). Sometimes, tracheotomy is urgently and very necessary to relieve severe airway stenosis firstly so as to maintain the patient’s vital signs. This is because some patients admitted to the hospital due to severe airway obstruction (17, 18).

Chu (5) found that radical surgical resection of PSCCT combined with adjuvant chemoradiotherapy results in a favorable outcome. As reported, for inoperable PSCCT patients or who declining surgery, chemotherapy (carboplatin and paclitaxel, cisplatin and docetaxel, paclitaxel) and radiotherapy is effective supplementary treatment options (7, 14, 21). After comprehensively reviewing the literature on PSCCT in recent years, it is found that treatment methods mainly based on surgery can improve the survival rate (Table 1). Besides, there is no evidence that oral supplementation with exogenous thyroxine is beneficial for the prognosis of thyroid squamous cell carcinoma because the tumor cell does not have the TSH receptor (22). Anlotinib combined with Sintilimab and other BRAF and MEK inhibitors (dabrafenib and trametinib) are reported effective with initial relief of PSCCT (18, 23).

**Table 1:** Characteristics and survival of primary squamous cell carcinoma of the thyroid in limited studies and literature.

Reference	No. of cases	Tumor	Tumor size	Invasion pattern	Survival
Zhang et al. (24)	1	PSCCT+PTC	2 cm	Trachea, esophagus	Alive
Li et al. (25)	1	PSCCT+follicular carcinoma	3.3 cm	Internal jugular vein, common carotid artery	7 months
Liu et al. (23)	1	PSCCT	7.8 cm	Trachea	5 months
Iwaki et al. (7)	1	PSCCT	3.8 cm	Esophagus, trachea	Alive
Brandenburg et al. (18)	1	PSCCT	5.4 cm	Larynx, trachea, esophagus	20 months
Xin et al. (3)	1	PSCCT	4 cm	Lung, face	< 1 month
Sun et al. (26)	1	PSCCT	3.5 cm	Internal jugular vein, sternocleidomastoid muscle	Alive

However, surgery and postoperative adjuvant treatment methods of PSCCT still lack a large amount of evidence-based medical support. Further research is required to explore additional treatment options and improve the prognosis for PSCCT.

## Conclusion

PSCCT is a rare disease with a lack of consensus on management typically diagnosed at an advanced stage with a poor prognosis. Clinical presentation, medical history, imaging examination, and especially immunohistochemistry are essential for accurate diagnosis of PSCCT. Although surgery is the preferred option, it may not always be curative due to the high local progression and metastasis rate. The management of PSCCT requires a multi-disciplinary approach. Further research works of deeper insights into various aspects need to be done.

## Data Availability

The original contributions presented in the study are included in the article/Supplementary material, further inquiries can be directed to the corresponding author.

## Supplementary material

SUPPLEMENTARY FIGURE S1 A timeline of patient’s diagnosis and treatment process.
