# Supplementary figures and images for: Primary thyroid squamous cell carcinoma with severe respiratory stenosis and endotracheal invasion: a case report with literature review
Source: Front Med (Lausanne). 2025 Jul 25;12:1631714. doi: 10.3389/fmed.2025.1631714 (PMC12331759; doi:10.3389/fmed.2025.1631714)

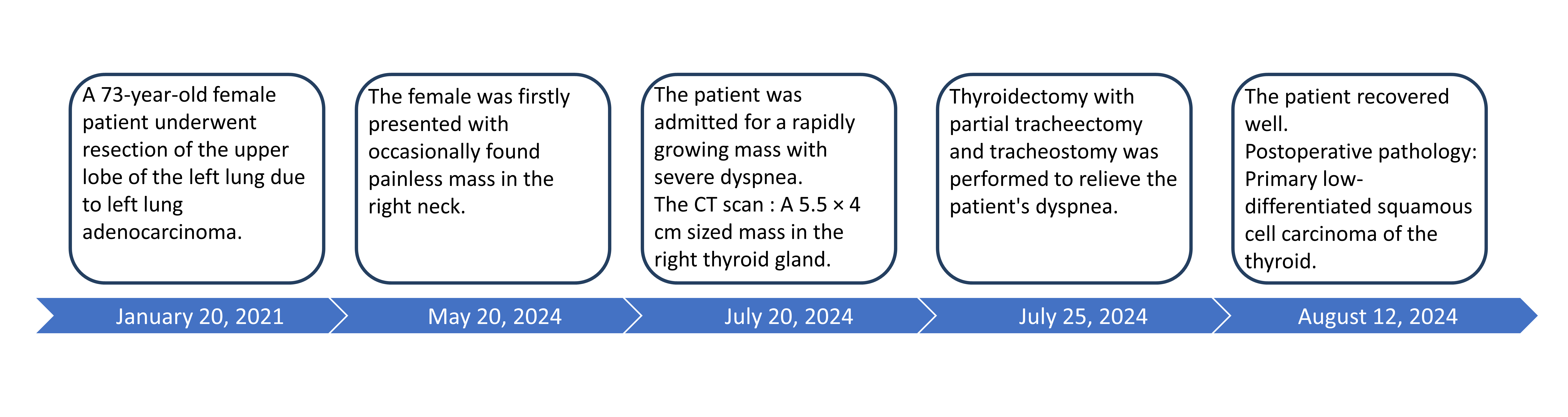

Supplement: SUPPLEMENTARY FIGURE S1 — A timeline of patient’s diagnosis and treatment process. [file Image_1.tif]
